# The Hospital for Diseases of the Skin, Blackfriars

**Published:** 1909-03-13

**Authors:** 


### Editor's Letter-Box.

### THE HOSPITAL FOR DISEASES OF THE SKIN, BLACKFRIARS.

We have received the following letter signed by Mr. William O'Malley, chairman of the above hospital. Several of the statements do not agree with the facts supplied to us, but as some are now *sub judice* we refrain from comment for the present :—

"I much regret to find that in your issue of the 6th February, 1909, you have made several statements in regard to the Hospital for the Diseases of the Skin, Blackfriars, which are not founded on fact.

"Firstly.—In the first place I may mention that your article of the 20th April, 1907, did not come to my knowledge until I saw it mentioned in your article of the 6th February, 1909. For more reasons than one it is not desirable at the present moment to discuss your previous article. Suffice it to say that the present committee believe that every act of theirs has been in perfect order and done with the best intentions and in the best interests of the hospital. Whether or not technical objections can be made to the appointment of either committee or trustees is a question which will be settled elsewhere, and is not one which can, for reasons known to you, be discussed in your columns. The present committee court the fullest investigation, and have nothing to fear in the shape of fair criticism upon their conduct of the affairs of the Hospital. They feel that you, relying no doubt upon inaccurate information, have gone somewhat beyond the bounds of that fair criticism which they have a right to expect from such a publication as yours and at the same time express your regret at having been misled as to facts. I feel sure that your desire is the same as that of the present committee—namely, that the hospital shall be placed upon a thoroughly satisfactory basis, and that its rules and regulations (for which the present committee is not responsible) shall be unexceptionable. I will now deal in detail with your article of the 6th February, 1909.

"It is not true that the senior surgeons both resigned in protest against the proceedings of the committee mentioned by you. The proceedings which it was alleged were objected to by the surgeon took place on the 26th March, 1907. One of these surgeons resigned on the 18th October, 1907, but continued to render service and acted on the committee until December 1907, and parted with the committee on perfectly amicable terms. The other surgeon did not resign until 12th January, 1909.

"Secondly.—The statement that the entire medical staff of the hospital consisted of the former assistant surgeon is without foundation. Since the date in question—namely, December, 1907—the surgeon, who was the late Mr. Thomas Joseph Patrick Hartigan, acted in conjunction with the surgeon (who retired on the 12th January, 1909), and immediately on such retirement steps were taken for the appointment of a surgeon to fill his place. The two surgeons referred to were assisted by four fully qualified clinical assistants, and these four clinical assistants are still working at the hospital. Mr. Hartigan died, as stated, on the 25th January, 1909, being a fortnight after his colleague's retirement. His death was a loss to Science and the Hospital. Eulogistic references to him have appeared in the public Press. He was the pioneer of the therapeutic use of radium, and great success followed his application of Catephoresis to Lupus Erythematosus. He was one of the most prominent members of the Council of the Dermatological Society, and most of his original work is recorded in the transactions of that body and those of the Royal Society of Medicine. On his death the committee immediately communicated with and obtained the assistance of Mr. Willmott Evans, who has since been appointed as assistant surgeon to the hospital and is assisted by the four clinical assistants aforesaid. The appointment of another surgeon or of a physician has not yet been made.

"Thirdly.—You also insinuated that Messrs. Cooper, in consequence of the proceedings referred to, had declined to be further identified with the affairs of the hospital. This is not the fact. Messrs. Cooper, the well-known accountants, considered that they had rendered sufficient, almost entirely unrecompensed, service to the hospital during the 40 years that they had been connected with it, and that an opportunity should be given now for another firm of accountants to carry on the work, and they have emphatically stated that the reason you give for their retirement is not correct.

"Fourthly.—You also insinuated that those responsible for the administration of the King Edward's and the Sunday Hospital funds had refused grants to the hospital. There is no foundation for either suggestion. No application was made to either fund for assistance, as the income of the hospital has sufficed to meet its expenditure. The past year has been, as far as is known, the most successful year in the history of the hospital, and this was chiefly owing to the great interest the late Mr. Hartigan took in the affairs and to greatly improved management in every department. The fact that grants were not made from the two funds is not to be taken to be a point against the hospital any more than it would be a point against St. Thomas's or St. George's Hospitals, neither of which received a grant during the past year. The question of the rules of the hospital and the trustees is receiving the fullest consideration, but for the reason I have given I cannot comment upon what is being done in that direction.

"A leaflet containing your article of the 6th February, 1909, has been printed and widely circulated amongst subscribers to the hospital and amongst medical men generally, and I am entitled to ask you to assist me in making this letter of mine in answer as widely known as was the article to which I have taken exception.

"February 26th, 1909."

